# Fisetin Suppresses the Proliferation and Metastasis of Renal Cell Carcinoma through Upregulation of MEK/ERK-Targeting CTSS and ADAM9

**DOI:** 10.3390/cells8090948

**Published:** 2019-08-21

**Authors:** Min-Hong Hsieh, Jen-Pi Tsai, Shun-Fa Yang, Hui-Ling Chiou, Chia-Liang Lin, Yi-Hsien Hsieh, Horng-Rong Chang

**Affiliations:** 1 Institute of Medicine, Chung Shan Medical University, Taichung 40201, Taiwan; 2 Department of Orthopedics, Dalin Tzu Chi Hospital, Buddhist Tzu Chi Medical Foundation, Chiayi 62247, Taiwan; 3 School of Medicine, Tzu Chi University, Hualien 97071, Taiwan; 4 Division of Nephrology, Department of Internal Medicine, Dalin Tzu Chi Hospital, Buddhist Tzu Chi Medical Foundation, Chiayi 62247, Taiwan; 5 School of Medical Laboratory and Biotechnology, Chung Shan Medical University, Taichung 40201, Taiwan; 6 Department of Biochemistry, School of Medicine, Chung Shan Medical University, Taichung 40201, Taiwan; 7 Clinical Laboratory, Chung Shan Medical University Hospital, Taichung 40201, Taiwan; 8 School of Medicine, Chung Shan Medical University, Taichung 40201, Taiwan; 9 Division of Nephrology, Department of Medicine, Chung Shan Medical University Hospital, Taichung 40201, Taiwan

**Keywords:** fisetin, CTSS, ADAM9, renal cell carcinoma, proliferation, migration, invasion

## Abstract

Fisetin, a natural flavonoid, is known to have anticarcinogenic effects against several cancers, but its role in mediating renal cell carcinoma (RCC) progression has not been delineated. Cell viability, cytotoxicity, and cell cycle distribution were measured using the 3-(4,5-cimethylthiazol-2-yl)-2,5-diphenyl tetrazolium bromide assay and propidium iodide staining with flow cytometry. The in vitro migration and invasion assay was used to examine in vivo cell migration and invasion. Human protease antibody array analysis was conducted with cell migration/invasion-related proteins. Western blotting and quantitative reverse transcription polymerase chain reaction were used for assessing protein expression related to the cell cycle, cell invasion, and mitogen-activated protein kinase (MAPK) signaling pathway. We found that fisetin significantly inhibited cell viability through cell cycle arrest in the G2/M phase, in addition to downregulating cyclin D1 and upregulating p21/p27. Fisetin inhibited the migration and invasion of human RCC cells through the downregulation of CTSS and a disintegrin and metalloproteinase 9 (ADAM9). Fisetin also upregulated ERK phosphorylation in 786-O and Caki-1 cells. Furthermore, treatment with a MEK inhibitor (UO126) reduced the inhibitory effects of fisetin on the metastasis of RCC cells through the ERK/CTSS/ADAM9 pathway. Fisetin inhibits proliferation and metastasis of RCC cells by downregulating CTSS and ADAM9 through the MEK/ERK signaling pathway. These findings indicate that fisetin is a promising antitumor agent against RCC.

## 1. Introduction 

Renal cell carcinoma (RCC) is the most lethal genitourinary cancer in patients with advanced or metastatic RCC, leading to a poor prognosis [1]. In the absence of distant metastasis, nephrectomy can be used for RCC treatment; however, if metastasis is present, immunotherapeutic agents in addition to nephrectomy are needed to increase the median survival time [2]. Although recently developed targeted therapies with tyrosine kinase and mTOR inhibitors are beneficial for overcoming the resistance of RCC to chemotherapy, radiotherapy, and hormone therapy, the prognosis remains poor for advanced or metastatic RCC [3,4]. 

Evidence indicates that natural food and herbal medicine may play promising roles in RCC treatment [5,6]. Fisetin (3,3′,4′,7-tetrahydroxyfavone), a naturally occurring flavonoid commonly found in plants, is effective against cancer, and its possible mechanisms include inhibition of proliferation, angiogenesis, induction of cell cycle arrest, and reversal of multidrug resistance [7]. Fisetin has therapeutic effects against hepatoma, lung adenocarcinoma, non-small-cell lung cancer, and prostate cancer through the induction of apoptosis or the inhibition of invasion or migration [8,9,10,11]. Through the modulation of the caspase-dependent pathway and the interruption of the p38 mitogen-activated protein kinase (MAPK)-dependent pathway, we found that fisetin can induce apoptosis and inhibit the migration and invasion of human cervical cancer cells [12,13]. 

Cysteine cathepsin proteases, including 11 members which were originally known as degradative enzymes of the lysosome, are frequently dysregulated in malignant transformation and induce tumor progression or proliferation, degradation of the extracellular matrix (ECM), invasion, and metastasis in numerous cancers through various mechanisms [14,15]. The upregulation of cathepsin B (CTSB), cathepsin S (CTSS), and cathepsin V (CTSV) is correlated with tumor progression and poor prognosis and is a potential predictor of response to chemotherapy [16,17,18]. A disintegrin and metalloproteinase (ADAM), a member of the metzincin superfamily of matrix metalloproteinases (MMPs), is involved in numerous biological functions, including the adhesion, migration, proteolysis, and malignant transformation of various cancers [19,20,21]. The upregulation of specific ADAMs in advanced and malignant human cancers indicates its modulating role in the progression of cancer, such as the promotion of prostate intraepithelial neoplasia or colon cancer invasion [20,22]. 

Despite encouraging reports regarding the efficacy of new RCC therapies [3,4], the overall survival time for patients with advanced or metastatic RCC remains short. In this study, using the human protease array, we attempted to demonstrate the correlation between RCC and cathepsins and ADAMs, as well as the inhibitory effects of fisetin for CTSB, CTSS, and ADAM9. Fisetin is effective against cancer without causing the side effects induced by traditional therapeutic agents. Furthermore, no study has evaluated the effects of fisetin on RCC. Therefore, we conducted this study to clarify the effect of fisetin on RCC and to investigate the mechanism through which fisetin acts on highly invasive human RCC cells. 

## 2. Materials and Methods

### 2.1. Chemical, Reagents, and Antibody

Fisetin, 3-(4,5-cimethylthiazol-2-yl)-2,5-diphenyl tetrazolium bromide (MTT), and propidium iodide were purchased from Sigma-Aldrich (St. Louis, MO, USA). Antibodies against ADAM9 and p-ERK were purchased from Cell Signaling Technology (Danvers, MA, USA). U0126 (an MEK1/2 inhibitor), and antibodies against p-p38, t-p38, t-ERK, p-JNK, t-JNK, CTSS, CTSB, CTSV, p21, p27, cyclin B1, and β-actin were purchased from Santa Cruz Biotechnology (Santa Cruz, CA, USA). The human protease array kit (Catalog#ARY021), recombinant human ADAM9 protein (Rh-ADAM9), and recombinant human CTSS protein (Rh-CTSS) were purchased from R&D Systems (Minneapolis, MN, USA).

### 2.2. Cell Culture

The human RCC cell line 786-O was cultured in RPMI medium; CaKi-1, ACHN, and A-498 were routinely cultured in MEM medium. Each growth medium was supplemented with 10% fetal bovine serum and contained 100 U/mL penicillin, 0.1 mM NEAA, and 1 mM sodium pyruvate. The cultures were incubated at 37 °C in a humidified atmosphere of 5% CO_2_.

### 2.3. MTT Assay

The effect of fisetin on cytotoxicity was determined using the MTT assay. RCC cells (1 × 10^4^ cells/well) were seeded into a 24-well plate. The next day, they were treated with different concentrations of fisetin (20, 40, and 60 μM) for 24 h, after which the culture medium was removed and the MTT agents (0.5 mg/mL) were added. The mixture was then incubated for an additional 4 h. After incubation, the MTT agents were removed and 1 mL isopropanol was added to dissolve the purple crystal. The produced formazan was detected by measuring the absorbance at 570 nm using a microplate reader (Labsystems, Helsinki, Finland). The viability of the treated cells is presented as a percentage of vehicle-treated control cells. Three independent experiments were performed for statistical analysis.

### 2.4. Cell Toxicity Assay

A CCK8 kit assay (Abcam, Cambridge, MA, USA) was used to measure the cytotoxic effects of fisetin on human RCC cells. Human RCC cells (2–3 × 10^3^/well) were seeded into 96-well plates and treated with different concentrations of fisetin (20, 40, and 60 μM) for 24 h. Subsequently, the CCK-8 reagent was added to each well and incubated at 37 °C for 2 h. The absorbance wavelength was measured at 570nm by a microplate reader (Labsystems, Helsinki, Finland).

### 2.5. Colony Formation Assay

A colony formation assay was performed to assess the dimensions and numbers of colonies. The cells were seeded (1 × 10^3^ cells/well, depending on the cell type) into 6-well plates. After being treated with different concentrations (20, 40, and 60 μM) of fisetin for 7 days, the fisetin-containing medium was removed and the cells were washed with PBS twice. The colonies were fixed with 70% methanol and stained with 0.5% crystal violet. The images of stained colonies were captured using a Nikon photo camera.

### 2.6. Evaluation of Cell Cycle

786-O and CaKi-1 cells were plated at a density of 4 × 10^5^ cells on 6 cm dishes and then incubated with 0, 20, 40, and 60 μM fisetin for 24 h. After incubating for 24 h, cells were washed three times with PBS and fixed in 70% ethanol at −20 °C for 3 days. The fixed cells were stained with a PI reagent (50 μg/mL) and RNase A (1 mg/mL) for 15 minutes. The cell cycle distribution was detected and analyzed using a Muse Cell Analyzer (Millipore) Vantage flow cytometer.

### 2.7. Annen V/PI Staining by Flow Cytomrtey

Cells were treated with different concentrations of fisetin for 24 h, then collected and washed in cold PBS buffer. Apoptotic cells were measured using the Muse™ Annexin V and Dead Cell Kit (Part No. 4700-1485) according to the manufacturer’s instructions. Apoptotic cells were then quantified and analyzed using a Muse Cell Analyzer (Millipore, Darmstadt, Germany).

### 2.8. Migration and Invasion Assay

The migration assay was performed using Transwell inserts (Costar, NY, USA; 8 mm pore size) in 24-well dishes. For the invasion assay, the membrane was coated with a layer of Matrigel (BD Biosciences, Bedford, MA, USA) according to the manufacturer’s instructions. The following procedures were the same for both migration and invasion assays. After treatment with fisetin for 24 h, the cells were harvested and seeded into Transwell inserts at 1 × 10^4^ cells/well in serum-free medium and then incubated for 24 hat 37 °C in 5% CO_2_. Four random fields of each membrane were chosen to be photographed and counted by an inverted phase-contrast microscope. The migrated cells number = (the number of fisetin − treated cells/the number of control cells) × 100%.

### 2.9. Proteome Profiler Human Protease Array

A Human Protease Array Kit (R&D Systems, Minneapolis, MN, USA) was used as previously reported [23]. 786-O and Caki-1 cells were treated with fisetin (0, 60 μM) for 24 h, then lysed in the lysis buffer (R&D Systems). The nitrocellulose membranes, including 34 different capture antibodies, were blocked and washed for 10 mins, then incubated with Streptavidin-HRP in the array buffer. The signal was visualized by an enhanced chemiluminescence analysis system (Fuji Film, Tokyo, Japan).

### 2.10. Western Blot Analysis

786-O and Caki-1 cells were treated with various doses of fisetin (0, 20, 40, and 60 μM) for 24 h. Equal amounts of protein extracts (25 μg) were separated on 10% sodium dodecyl sulphate polyacrylamide gel electrophoresis and transferred onto polyvinylidene difluoride membranes. After blocking the membranes with 5% milk, the blots were incubated with primary antibodies in TBST buffer at 4 °C overnight. Western blotting bands were detected using the Western-HRP substrate (Millipore, Billerica, MA, USA), and the bands were visualized and detected using a Luminescent Image Analyzer (LAS-4000).

### 2.11. Quantitative Reverse Transcription Polymerase Chain Reaction

Cathepsin S and Cathepsin B mRNA levels were determined through quantitative reverse transcription polymerase chain reaction (qRT-PCR). The total RNA was extracted from the cells by using TRIzol reagent. cDNA was reverse transcribed from the total RNA by using the GoScriptTM Reverse Transcription Mix (Promega, MW, USA). GAPDH and Cathepsin S and Cathepsin B mRNA levels were determined using the GoTaq qPCR Master Mix (Promega, MW, USA). Primer sequences were as follows: GAPDH, 5′-GATCATCCCTGCCTCTACTG-3′ and 5′-GCCTGCTTCACCACCTTC-3′; Cathepsin B, 5′-AGAGTTATGTTTACCGAGGACCT-3′ and 3′-GATGCAGATCCGGTCAGAGA-5′; and Cathepsin S, 5′-GCCTGATTCTGTGGACTGG-3′ and 3′-GATGTACTGGAAAGCCGTTGT-5′. Cathepsin V, 5′-TCGCGTCCTCAAGGCAATC-3′ and 5′-CACAGTTGCGACTGCTTTCAT-3′, ADAM9, 5′-GCTAGTTGGACTGGAGATTTGG-3′ and 5′-TTATTACCACAGGAGGGAGCAC-3′. The fold change in relative mRNA expression was determined using the 2^−ΔΔCt^ method on a 7500 Real-Time PCR system (Applied Biosystems by Life Technologies).

### 2.12. Statistical Analysis

GraphPad Prism 5 was used for statistical analysis. Data are represented as the mean ± SEM of three independent experiments. Differences between groups were assessed through one-way analysis of variance. A *p* value of <0.05 was considered statistically significant.

## 3. Results

### 3.1. Fisetin Decreased RCC Cell Viability

**Figure 1:**
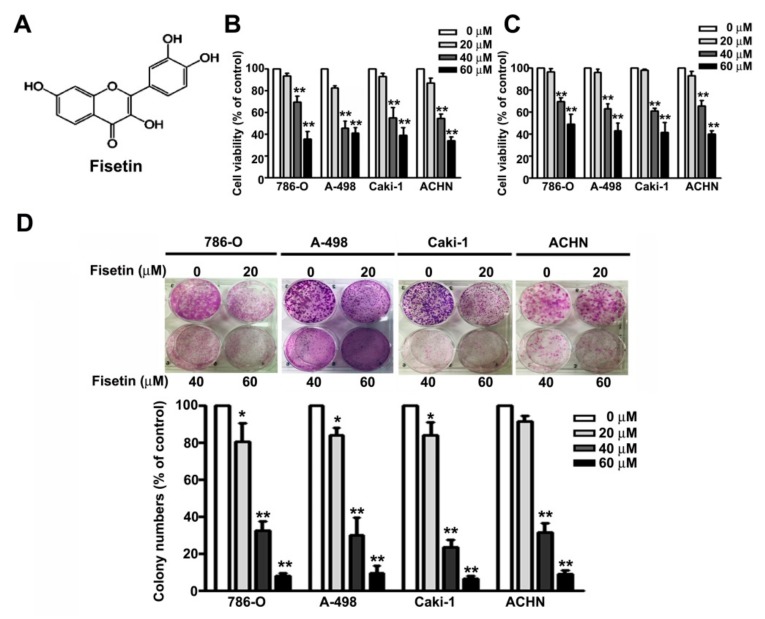
Fisetin inhibits the cell proliferation and colony formation ability of renal cell carcinoma (RCC) cell lines. (**A**) The chemical structures of fisetin. 786-O, A-498, Caki-1, and ACHN cells incubated with various concentrations (0, 20, 40, and 60 μM) of fisetin for 24 h. Cell viability was determined through the (**B**) 3-(4,5-cimethylthiazol-2-yl)-2,5-diphenyl tetrazolium bromide (MTT) assay and (**C**) CCK8 assay. (**D**) RCC cells were then harvested to determine the number of colonies after treatment with fisetin for 7 days. Bars show the value as the mean ± SE from three independent experiments. * *p* < 0.05, ** *p* < 0.01 compared with the untreated control (0 μM).

The fisetin structure is shown in Figure 1A. We first determined the cytotoxic effects of fisetin on RCC cell lines (786-O, A-498, Caki-1, and ACHN cells) through the MTT assay and CCK8 assay. We found that treating the RCC cells (786-O, A-498, Caki-1, and ACHN) with increasing concentrations (0, 20, 40, and 60 μM) of fisetin for 24 h significantly decreased cell viability in a dose-dependent manner (Figure 1B), similar to the results of the CCK8 assay (Figure 1C). The colony formation assay revealed that fisetin significantly reduced the colony formation of these cells in a dose-dependent manner (Figure 1D). Fisetin concentrations of 0–60 μM were used for further in vitro experiments.

### 3.2. Fisetin Induced Cell Cycle Arrest in the G2/M Phase and Assessment of Related G2/M Proteins of RCC Cells

**Figure 2:**
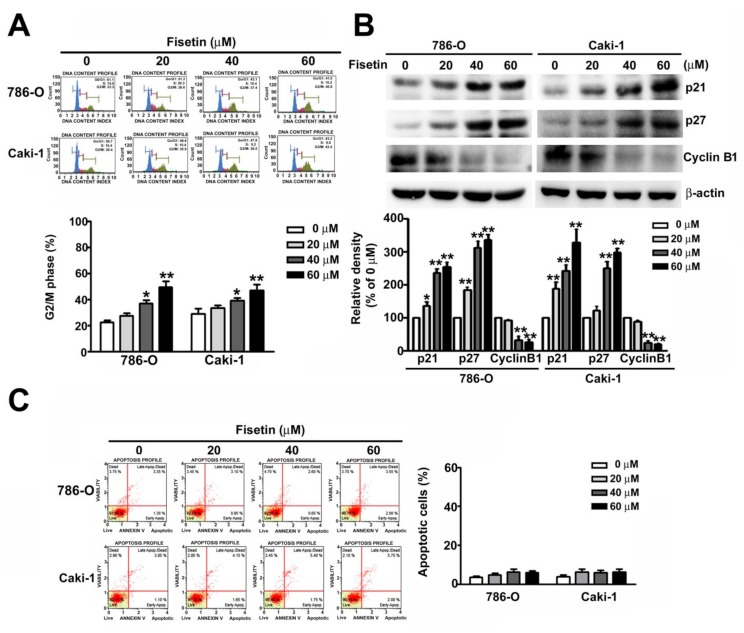
Fisetin-induced cell cycle arrest in the G2/M phase and assessment of relative G2/M protein expression. (**A**) Cell cycle analysis of 786-O and Caki-1 cells treated with various concentrations (0, 20, 40, and 60 μM) of fisetin. The cell cycle distribution was measured through flow cytometry. (**B**) The expression of G2/M-related proteins (cyclin D1, p21, and p27) was measured through Western blotting. (**C**) Cell apoptosis was detected with Annexin V/PI staining by flow cytometry. * *p* < 0.05, ** *p* < 0.01, compared with the untreated control (0 μM).

To explore the mechanism involved in fisetin-induced inhibition of RCC cell proliferation, the effects of fisetin on the cell cycle arrest were examined. 786-O and ACHN cells were incubated with various concentrations (0, 20, 40, and 60 μM) of fisetin for 24 h. The G2/M phase arrest increased from 35.5% to 46.9% and 41.5% to 53.6% in 786-O and Caki-1 cells, respectively, in a dose-dependent manner (Figure 2A). Furthermore, we assessed G2/M-related proteins from 786-O and CaKi-1 cells because of their relationship with the cell cycle. These results showed that the upregulation of p21 and p27 downregulated cyclin B1 in both fisetin-treated cell types (Figure 2B). Thus, fisetin inhibits 786-O and CaKi-1 cell proliferation, as well as arrests, then cells cycle in the G2/M phase.

### 3.3. Fisetin Inhibited Migration and Invasion of RCC Cells

**Figure 3:**
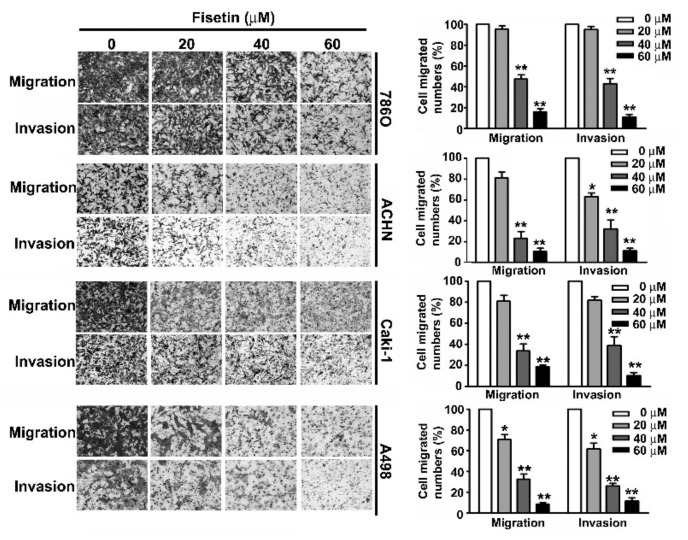
Fisetin inhibits cell migration and invasion of RCC cell lines. RCC cells were incubated with various concentrations (0, 20, 40, and 60 μM) of fisetin. The migration and invasion abilities were determined using a migration assay and Matrigel invasion assay. The cells in the lower surface of the Borden chamber were stained and photographed under a light microscope at 400× magnification. The quantification of migration and invasion abilities are shown as a histogram chart. Data are presented as the mean ± SE of at least three independent experiments. * *p* < 0.05, ** *p* < 0.01 compared with the untreated control (0 μM).

A crucial characteristic of metastasis is the migration and invasion of tumor cells [24]. Treating 786-O, A-498, Caki-1, and ACHN cells with various concentrations (0, 20, 40, and 60 μM) of fisetin for 24 h showed that fisetin inhibited the migration and invasion of these RCC cells in a dose-dependent manner, especially at a concentration of more than 40 μM (Figure 3).

### 3.4. Fisetin Inhibited CTSB, CTSS, and ADAM9 in RCC Cells

**Figure 4:**
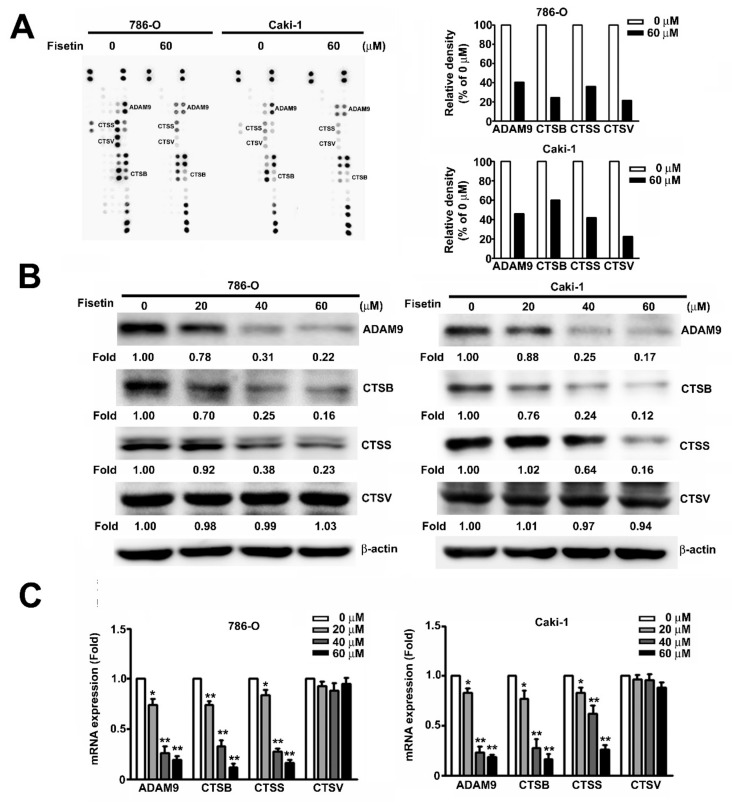
Effects of fisetin on the expression of CTSB, CTSS, CTSV, and a disintegrin and metalloproteinase (ADAM)9 in RCC cells. (**A**) A human protease array profile was performed on whole cell lysate from 786-O cells and then incubated with 0 and 60 μM fisetin for 24 h. Spots for CTSB, CTSS, CTSV, and ADAM9 were marked. The right panel shows the quantification of individual spot intensities compared with the control (0 μM). (**B**) 786-O cells were incubated with various concentrations (0, 20, 40, and 60 μM) of fisetin, and total cell lysates were then analyzed through Western blotting to determine the expression of CTSB, CTSS, CTSV, and ADAM9 proteins. β-actin was used as an internal control for protein, with equal loading. (**C**) The expression of CTSB, CTSS, CTSV, and ADAM9 mRNA was verified using qRT-PCR. Data are presented as the mean ± SE of at least three independent experiments. * *p* < 0.05, ** *p* < 0.01 compared with the untreated control (0 μM).

The human protease array kit assay was used to determine the possible mechanisms involved in the fisetin-induced inhibition of the migration and invasion of RCC cells. After incubating the cells with 0 and 60 μM fisetin for 24 h, spots of CTSB, CTSS, CTSV, and ADAM9 were marked; the expression of individual proteases significantly decreased after treatment with 60 μM fisetin (Figure 4A). Furthermore, protease array, Western blot, and qRT-PCR analyses were used to examine the protein and mRNA expression of ADAM9, CTSS, and CTSB after fisetin treatment. In 786-O and Caki-1 cells, ADAM9, CTSS, and CTSB expression showed a marked dose-dependent decrease after fisetin treatment. Only CTSV protein expression remained unchanged (Figure 4B,C). These results indicate that fisetin significantly inhibits the migration and invasion of RCC cells through the suppression of CTSB, CTSS, and ADAM9.

### 3.5. Upregulation of ERK Activation in Fisetin-Treated RCC Cell Migration and Invasion

**Figure 5:**
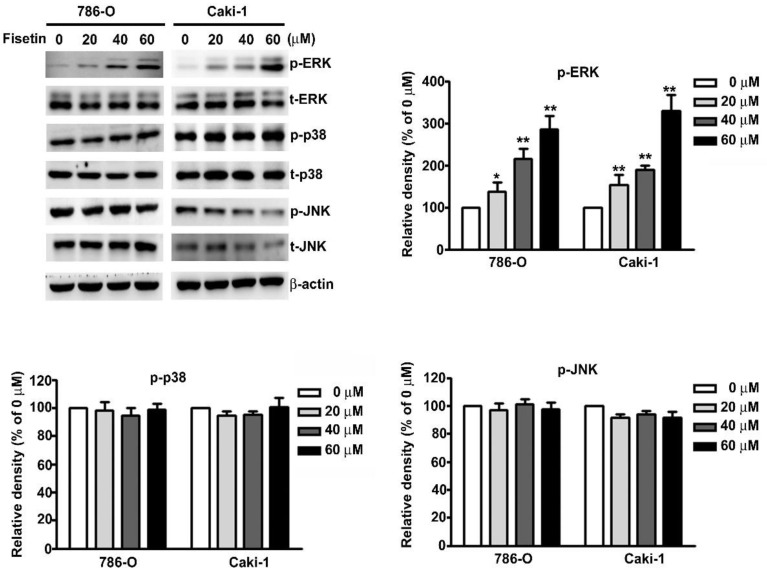
Effects of fisetin on the mitogen-activated protein kinase (MAPK) signaling pathway in RCC cell lines. 786-O and Caki-1 cells were incubated with various concentrations (0, 20, 40, and 60 μM) of fisetin, and total cell lysates were then analyzed through Western blotting to determine the protein expression levels of p-ERK, t-ERK, p-p38, t-p38, p-JNK, and t-JNK. β-actin was used as an internal control for protein, with equal loading. Data are presented as the mean ± SE of at least three independent experiments. * *p* < 0.05, ** *p* < 0.01 compared with the untreated control (0 μM).

MAPK signaling pathways have been known to be involved in the regulation of CTSS and ADAM9 expression in RCC cells [25,26]. We attempted to determine the signaling pathway involved in the fisetin-induced inhibition of CTSS and ADAM9 in 786-O and Caki-1 cells. To identify the underlying mechanisms involved in the fisetin-induced activation of the MAPK pathway, we treated 786-O and Caki-1 cells with various concentrations of fisetin. The results revealed a significant dose-dependent increase in ERK phosphorylation, whereas no significant differences were observed in the phosphorylation of p38 or JNK (Figure 5).

**Figure 6:**
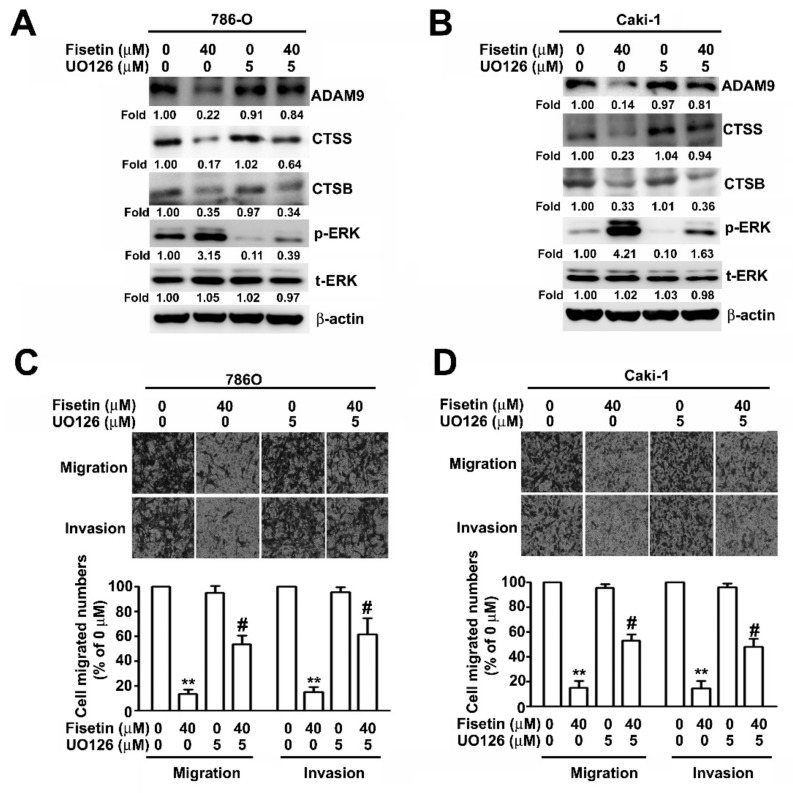
ERK pathways are involved in ADAM9 and CTSS regulation by fisetin. (**A**,**B**) 786-O and Caki-1 cells were pre-treated with UO126 (5 μM) for 2 h and then incubated with or without fisetin (40 μM) for 24 h. Cell lysates were subjected to Western blotting to determine the expression of ADAM9, CTSB, CTSS, p-ERK, and t-ERK proteins. β-actin was used as an internal control for protein, with equal loading. (**C**,**D**) Then cells were assessed for the abilities of migration and invasion. Data are presented as the mean ± SE of at least three independent experiments. ** *p* < 0.01 compared with the untreated control (0 μM). # *p* < 0.01, compared with fisetin treatment alone.

**Figure 7:**
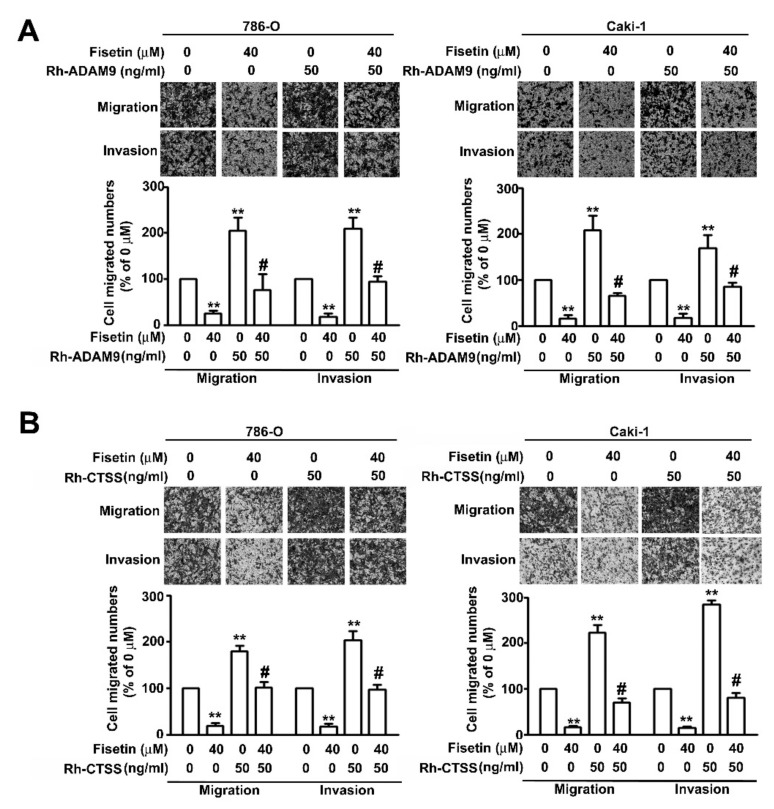
CTSS and ADAM9 are involved in the fisetin-induced inhibition of RCC cell migration and invasion. 786-O and Caki-1 cells were pretreated with (**A**) Rh-ADAM9 or (**B**) Rh-CTSS (50 ng/mL) for 2 h and then incubated with or without fisetin (40 μM) for 24 h. The migration and invasion abilities were analyzed through an in vitro migration and invasion assay. Data are presented as the mean ± SE of at least three independent experiments. ** *p* < 0.01 compared with the untreated control (0 μM). # *p* < 0.01, compared with fisetin treatment alone.

To further confirm that fisetin played a role in the inhibition of the migration and invasion of RCC cells through the activation of ERK signaling pathway, Western blotting was performed after pretreating 786-O and Caki-1 cells with an MEK inhibitor, UO126 (5 μM), for 2 h and incubating with fisetin (40 μM) for 24 h. It was revealed that treatment with the combination of fisetin with UO126 or with UO126 significantly downregulated ERK phosphorylation and upregulated CTSS and ADAM9 compared with treatment with fisetin alone; however, CTSB protein expression remained unchanged (Figure 6A). The migration and invasion assay revealed that treatment with the combination of fisetin with UO126 or with UO126 further enhanced cell migration and invasion compared with treatment with fisetin alone (Figure 6B). To confirm whether ADAM9 and CTSS are involved in the fisetin-induced inhibition of cell migration and invasion, 786-O and Caki-1 cells were pretreated with Rh-ADAM9 (50 ng/mL) or Rh-CTSS (50 ng/mL) for 2 h; then, fisetin (40 μM) was added to the medium and incubated for another 22 h. We found that fisetin combined with Rh-ADAM9 or Rh-CTSS significantly restored the migration and invasion of 786-O and Caki-1 cells compared with fisetin alone (Figure 7A,B). Thus, ADAM9 and CTSS play a crucial role in RCC cell migration and invasion. This result clearly indicates that fisetin inhibits CTSS and ADAM9 through the upregulation of the ERK signaling pathway, which plays a key role in the inhibition of the migration and invasion of RCC cells.

## 4. Discussion

**Figure 8:**
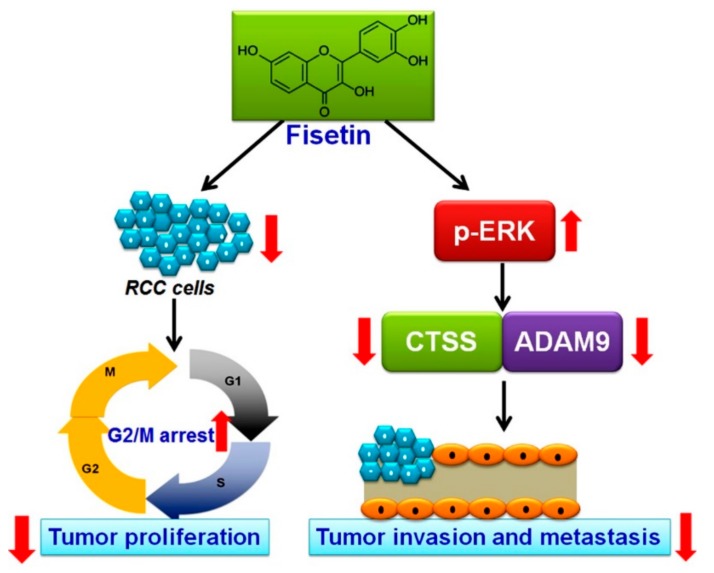
Schematic representation of the antiproliferative and antimetastatic effects of fisetin in human RCC cells. Fisetin has antiproliferative and antimetastatic effects on human RCC cells mediated by the downregulation of CTSS and ADAM9 through the ERK signaling pathway.

Although recent advances have improved the overall survival rate of patients with RCC, the prognosis is poor for advanced or distant metastases of RCC [1]. RCC management has evolved from nephrectomy and chemotherapy for localized RCC to targeted therapies against the vascular endothelium growth factor pathway or mTOR inhibitors for advanced or metastatic RCC. Because the available targeted therapies are expensive and have associated side effects [27], a new target agent should be identified for the better prognosis of patients with RCC. In this study, we found that (1) fisetin has cytotoxic effects on RCC cells and induces RCC cell cycle arrest in the G2/M phase; (2) fisetin inhibits the invasion and migration of highly invasive RCC cells in vitro; and (3) fisetin downregulates CTSS and ADAM9 through the upregulation of the ERK signaling pathway, leading to antimetastatic effects (Figure 8).

Fisetin is a naturally occurring flavonoid that has anticarcinogenic effects, such as antiproliferative, antioxidative, and antiangiogenic activities, and it promotes cell cycle arrest and apoptosis. [7,28]. The treatment of epidermoid carcinoma cells with fisetin resulted in a dose- and time-dependent decrease in colony formation and cell viability through the induction of apoptosis and cell cycle arrest in the G2/M phase [29]. Similarly, studies of non-small-cell lung cancer [8], pulmonary adenocarcinoma [9], hepatocellular carcinoma [10], and cervical cancer [13] showed that the cytotoxic effects of fisetin result from the production of reactive oxidative species as well as the induction of cell cycle arrest and apoptosis through the activation of the caspase cascade, the upregulation of proapoptotic-related proteins or p53, or the downregulation of antiapoptotic-related proteins. A previous study reported that, in RCC Caki-1 cells, fisetin induced apoptosis through the p53-mediated upregulation of DR5 [30]. Some evidence from in vitro studies has demonstrated that fisetin induces cell cycle arrest at the G2/M phase of HT29 cells and A431 cells through the downregulation of cyclin E and cyclin D1 and the upregulation of p21 expression [29,31]. Other studies have demonstrated that fisetin treatment of bladder T24 and prostate cancer LNCaP cells results in the induction of G0/G1 cell cycle arrest with increased expression levels of p21 and p27 [32,33]. A combination of etoposide and fisetin was found to inhibit human osteosarcoma cell proliferation and cell cycle arrest at the G2/phase, resulting in reduced expression levels of cyclin B1 and cyclin E [34]. Similarly, in the present study, we found that fisetin treatment in RCC cells significantly induced cell cycle arrest in the G2/M phase. 

Moreover, fisetin exerts its cytotoxic effects by modulating the MAPK signaling pathway, namely through the inhibition or activation of the pathway [9,13,35]. Conversely, the upregulation of ERK1/2 phosphorylation after treatment with fisetin in human cervical cancer HeLa cells led to apoptosis through the caspase-3/-8-dependent pathway [13]. In non-small-cell lung cancer or glioma cells, fisetin induced apoptosis or inhibited invasion and migration by activating the MAPK signaling pathway through ERK-mediated CHOP upregulation or ADAM9 downregulation [26,35]. Thus, similar to studies showing that the inappropriate function of ERK-mediated cascades could lead to aberrant cell survival, tumor invasion, and metastasis, we found that fisetin could suppress cell proliferation, invasion, and migration through the activation of the ERK signaling pathway. 

Cysteine cathepsin protease was originally known as a degradative enzyme that integrated into nearly all the processes of functions of lysosomes, such as protein degradation, autophagy, antigen presentation, and cell death; it was also frequently reported to be involved in malignant transformation, which included the alteration of adhesion, degradation of ECM for invasion, invasion into blood vessels, and establishment of a new tumor at distant sites [36,37]. Studies have demonstrated a strong correlation between CTSB activity and the aggressively metastatic potential of pancreatic cancer or melanoma [14,38]. For pancreatic cancer, CTSB and CTSS were found to have critical roles in promoting tumor growth, angiogenesis, and invasive abilities [37]. CTSS mediates the transmigration of the blood–brain barrier in breast cancer through the proteolysis of junction adhesion molecules [36]. Additionally, when CTSB, CTSS, and CTSV expression levels are significantly high, this could lead to a worse prognosis in colorectal cancer, squamous cell carcinoma of the lung, and breast cancer; thus, it may be a poor predictor of response to chemotherapy [16,17,18]. Recently, some studies have reported on the sustained activation of MEK/ERK to decrease ADAM9 expression and inhibit cell migration and invasion by galangin and Licochalcome A in human glioma cells [39,40]. ADAM9 was shown to be involved in invasion and distant metastasis in various cancers, and is correlated with adverse prognostic parameters and short patient survival time [41]. Thus, these studies emphasized the role of ADAM9 in various cancers and highlighted the association of ADAM9 with a poor RCC prognosis. However, the current study showed that sulforaphane or hispolon could similarly activate autophagy and suppress the invasion and metastasis of oral cancer cells or cervical cancer cells by downregulating CTSS through the upregulation of the MEK/ERK signaling pathway [42,43]. Consistent with the above studies, we were able to demonstrate that fisetin inhibited the metastatic ability of human RCC cells through the activation of the MEK/ERK pathways involved in CTSS/ADAM9 expression.

According to the MTT and CCK8 assay used to analyze the fisetin treatment of 24 h, fisetin inhibited cell viability on RCC cells; however, the inhibitory effects on migration and invasion may be due to the effects on cell viability. A number of research works have demonstrated that fisetin exhibits significantly inhibitory effects on tumor progression by inhibiting cell viability, cell cycle arrest, and the invasion process in melanoma [44] and prostate cancer [45]. Fisetin inhibits proliferation, as well as invasive and reverse epithelial-to-mesenchymal transition, by targeting the ERK and PTEN/Akt/GSK3β signaling pathways in human NSCLCs, such as A549 cells [9] and TNBC [46]. Based on these findings, which are similar to the results of the present study, we can conclude that fisetin inhibits cell migration and invasion due to its inhibitory effect on cell viability. According to this finding, coupled with the relationship between MEK/ERK signaling and tumor proliferation/invasion progression, we speculated that the anti-invasive effect of fisetin on RCC was mediated by targeting CTSS/ADAM9 expression, which is the most important key molecule for MEK/ERK activation. Another question is whether the MEK/ERK signaling pathways is involved in the fisetin-induced inhibition of cell proliferation. Unexpected results showed that fisetin combined with U0126 does not affect cell viability in 786-O and Caki-1 cells. Therefore, other signaling pathways may be involved in antiproliferative effect of fisetin in RCC cells, which will be the focus of future study.

To the best of our knowledge, this is the first study to demonstrate the antiproliferative and antimetastatic effects of fisetin in human RCC cells in vitro. We observed that fisetin not only has cytotoxic effects on RCC cells through G2/M phase arrest, but it also inhibits the migration and invasion of RCC cells by downregulating CTSS and ADAM9 through the ERK signaling pathway. Thus, fisetin is a promising antiproliferative and antimetastatic agent for the treatment of RCC. 

## References

[1] Znaor A., Lortet-Tieulent J., Laversanne M., Jemal A., Bray F. (2015). International Variations and Trends in Renal Cell Carcinoma Incidence and Mortality. Eur. Urol..

[2] Conti S.L., Thomas I.C., Hagedorn J.C., Chung B.I., Chertow G.M., Wagner T.H., Brooks J.D., Srinivas S., Leppert J.T. (2014). Utilization of cytoreductive nephrectomy and patient survival in the targeted therapy era. Int. J. Cancer.

[3] Siegel R., Naishadham D., Jemal A. (2013). Cancer statistics, 2013. CA Cancer J. Clin..

[4] Chiong E., Tay M.H., Tan M.H., Kumar S., Sim H.G., Teh B.T., Umbas R., Chau N.M. (2012). Management of kidney cancer in Asia: resource-stratified guidelines from the Asian Oncology Summit 2012. Lancet Oncol..

[5] Chen C.-M., Hsieh S.-C., Lin C.-L., Lin Y.-S., Tsai J.-P., Hsieh Y.-H. (2017). Alpha-Mangostin Suppresses the Metastasis of Human Renal Carcinoma Cells by Targeting MEK/ERK Expression and MMP-9 Transcription Activity. Cell. Physiol. Biochem..

[6] Liu C., Lee W.-C., Huang B.-M., Chia Y.-C., Chen Y.-C., Chen Y.-C. (2017). 16-Hydroxycleroda-3, 13-dien-15, 16-olide inhibits the proliferation and induces mitochondrial-dependent apoptosis through Akt, mTOR, and MEK-ERK pathways in human renal carcinoma cells. Phytomedicine.

[7] Ren W., Qiao Z., Wang H., Zhu L., Zhang L. (2003). Flavonoids: Promising anticancer agents. Med. Res. Rev..

[8] Kang K.A., Piao M.J., Hyun J.W. (2015). Fisetin induces apoptosis in human nonsmall lung cancer cells via a mitochondria-mediated pathway. In vitro cellular & developmental biology. Animal.

[9] Liao Y.-C., Shih Y.-W., Chao C.-H., Lee X.-Y., Chiang T.-A. (2009). Involvement of the ERK Signaling Pathway in Fisetin Reduces Invasion and Migration in the Human Lung Cancer Cell Line A549. J. Agric. Food Chem..

[10] Chen Y.-C., Shen S.-C., Lee W.-R., Lin H.-Y., Ko C.-H., Shih C.-M., Yang L.-L. (2002). Wogonin and fisetin induction of apoptosis through activation of caspase 3 cascade and alternative expression of p21 protein in hepatocellular carcinoma cells SK-HEP-1. Arch. Toxicol..

[11] Khan N., Adhami V.M., Mukhtar H. (2010). Apoptosis by dietary agents for prevention and treatment of prostate cancer. Endocr. Relat. Cancer.

[12] Chou R.-H., Hsieh S.-C., Yu Y.-L., Huang M.-H., Huang Y.-C., Hsieh Y.-H. (2013). Fisetin Inhibits Migration and Invasion of Human Cervical Cancer Cells by Down-Regulating Urokinase Plasminogen Activator Expression through Suppressing the p38 MAPK-Dependent NF-κB Signaling Pathway. PLoS ONE.

[13] Ying T.H., Yang S.F., Tsai S.J., Hsieh S.C., Huang Y.C., Bau D.T., Hsieh Y.H. (2012). Fisetin induces apoptosis in human cervical cancer HeLa cells through ERK1/2-mediated activation of caspase-8-/caspase-3-dependent pathway. Arch. Toxicol..

[14] Sloane B., Dunn J.R., Honn K. (1981). Lysosomal cathepsin B: Correlation with metastatic potential. Science.

[15] A Joyce J., Baruch A., Chehade K., Meyer-Morse N., Giraudo E., Tsai F.-Y., Greenbaum D.C., Hager J.H., Bogyo M., Hanahan D. (2004). Cathepsin cysteine proteases are effectors of invasive growth and angiogenesis during multistage tumorigenesis. Cancer Cell.

[16] Harbeck N., Alt U., Berger U., Krüger A., Thomssen C., Jänicke F., Höfler H., E Kates R., Schmitt M. (2001). Prognostic impact of proteolytic factors (urokinase-type plasminogen activator, plasminogen activator inhibitor 1, and cathepsins B, D, and L) in primary breast cancer reflects effects of adjuvant systemic therapy. Clin. Cancer Res..

[17] A Gormley J., Hegarty S.M., O’Grady A., Stevenson M.R., E Burden R., Barrett H.L., Scott C.J., A Johnston J., Wilson R.H., Kay E.W. (2011). The role of Cathepsin S as a marker of prognosis and predictor of chemotherapy benefit in adjuvant CRC: a pilot study. Br. J. Cancer.

[18] Werle B., Lötterle H., Schanzenbächer U., Lah T.T., Kalman E., Kayser K., Bülzebruck H., Schirren J., Krasovec M., Kos J. (1999). Immunochemical analysis of cathepsin B in lung tumours: an independent prognostic factor for squamous cell carcinoma patients. Br. J. Cancer.

[19] Sung S.-Y., Kubo H., Shigemura K., Arnold R.S., Logani S., Wang R., Konaka H., Nakagawa M., Mousses S., Amin M. (2006). Oxidative Stress Induces ADAM9 Protein Expression in Human Prostate Cancer Cells. Cancer Res..

[20] Mazzocca A., Coppari R., De Franco R., Cho J.-Y., Libermann T.A., Pinzani M., Toker A. (2005). A Secreted Form of ADAM9 Promotes Carcinoma Invasion through Tumor-Stromal Interactions. Cancer Res..

[21] O’Shea C., McKie N., Buggy Y., Duggan C., Hill A.D., McDermott E., O’Higgins N., Duffy M.J. (2003). Expression of ADAM-9 mRNA and protein in human breast cancer. Int. J. Cancer.

[22] Peduto L., Reuter V.E., Shaffer D.R., Scher H.I., Blobel C.P. (2005). Critical Function for ADAM9 in Mouse Prostate Cancer. Cancer Res..

[23] Chiang K.-C., Lai C.-Y., Chiou H.-L., Lin C.-L., Chen Y.-S., Kao S.-H., Hsieh Y.-H. (2019). Timosaponin AIII inhibits metastasis of renal carcinoma cells through suppressing cathepsin C expression by AKT/miR-129-5p axis. J. Cell. Physiol..

[24] Ricketts C.J., Linehan W.M. (2018). The origin, evolution and route to metastasis of clear cell RCC. Nature reviews. Nephrology.

[25] Hsieh M.J., Lin C.W., Chen M.K., Chien S.Y., Lo Y.S., Chuang Y.C., Hsi Y.T., Lin C.C., Chen J.C., Yang S.F. (2017). Inhibition of cathepsin S confers sensitivity to methyl protodioscin in oral cancer cells via activation of p38 MAPK/JNK signaling pathways. Sci. Rep..

[26] Chen C.M., Hsieh Y.H., Hwang J.M., Jan H.J., Hsieh S.C., Lin S.H., Lai C.Y. (2015). Fisetin suppresses ADAM9 expression and inhibits invasion of glioma cancer cells through increased phosphorylation of ERK1/2. Tumour Biol..

[27] Heng D.Y. (2016). The next 10 years: Challenges for the future and overcoming resistance to targeted therapies for renal cell carcinoma. Can. Urol. Assoc. J..

[28] Moon Y.J., Wang X., Morris M.E. (2006). Dietary flavonoids: Effects on xenobiotic and carcinogen metabolism. Toxicol. In Vitro.

[29] Pal H.C., Sharma S., Elmets C.A., Athar M., Afaq F. (2013). Fisetin inhibits growth, induces G2/M arrest and apoptosis of human epidermoid carcinoma A431 cells: Role of mitochondrial membrane potential disruption and consequent caspases activation. Exp. Dermatol..

[30] Min K.-J., Nam J.-O., Kwon T.K. (2017). Fisetin Induces Apoptosis Through p53-Mediated Up-Regulation of DR5 Expression in Human Renal Carcinoma Caki Cells. Molecules.

[31] Lu X., Jung J.I., Chun H.S., Kwon D.Y., Park J.H., Cho H.J., Lim D.Y., Lee H.S. (2005). Fisetin Inhibits the Activities of Cyclin-Dependent Kinases Leading to Cell Cycle Arrest in HT-29 Human Colon Cancer Cells. J. Nutr..

[32] Khan N., Afaq F., Syed D.N., Mukhtar H. (2008). Fisetin, a novel dietary flavonoid, causes apoptosis and cell cycle arrest in human prostate cancer LNCaP cells. Carcinogenesis.

[33] Li J., Cheng Y., Qu W., Sun Y., Wang Z., Wang H., Tian B. (2011). Fisetin, a dietary flavonoid, induces cell cycle arrest and apoptosis through activation of p53 and inhibition of NF-kappa B pathways in bladder cancer cells. Basic Clin. Pharmacol. Toxicol..

[34] Ferreira de Oliveira J.M.P., Pacheco A.R., Coutinho L., Oliveira H., Pinho S., Almeida L., Fernandes E., Santos C. (2018). Combination of etoposide and fisetin results in anti-cancer efficiency against osteosarcoma cell models. Arch. Toxicol..

[35] Kang K.A., Piao M.J., Hewage S.R.K.M., Ryu Y.S., Oh M.C., Kwon T.K., Chae S., Hyun J.W. (2016). Fisetin induces apoptosis and endoplasmic reticulum stress in human non-small cell lung cancer through inhibition of the MAPK signaling pathway. Tumor Boil..

[36] Sevenich L., Bowman R.L., Mason S.D., Quail D.F., Rapaport F., Elie B.T., Brogi E., Brastianos P.K., Hahn W.C., Holsinger L.J. (2014). Analysis of tumor- and stroma-supplied proteolytic networks reveals a brain metastasis-promoting role for cathepsin S. Nat. Cell Bio..

[37] Gocheva V., Wang H.-W., Gadea B.B., Shree T., Hunter K.E., Garfall A.L., Berman T., Joyce J.A. (2010). IL-4 induces cathepsin protease activity in tumor-associated macrophages to promote cancer growth and invasion. Genes Dev..

[38] Tzanakakis G.N., Margioris A.N., Tsatsakis A.M., Vezeridis M.P. (2003). The metastatic potential of human pancreatic cell lines in the liver of nude mice correlates well with cathepsin B activity. Int. J. Gastrointest. Cancer.

[39] Lei D., Zhang F., Yao D., Xiong N., Jiang X., Zhao H. (2018). Galangin increases ERK1/2 phosphorylation to decrease ADAM9 expression and prevents invasion in A172 glioma cells. Mol. Med. Rep..

[40] Huang C.-F., Yang S.-F., Chiou H.-L., Hsu W.-H., Hsu J.-C., Liu C.-J., Hsieh Y.-H. (2018). Licochalcone A inhibits the invasive potential of human glioma cells by targeting the MEK/ERK and ADAM9 signaling pathways. Food Funct..

[41] Fritzsche F.R., Wassermann K., Jung M., Tölle A., Kristiansen I., Lein M., Johannsen M., Dietel M., Jung K., Kristiansen G. (2008). ADAM9 is highly expressed in renal cell cancer and is associated with tumour progression. BMC Cancer.

[42] Chen C.-T., Hsieh M.-J., Hsieh Y.-H., Hsin M.-C., Chuang Y.-T., Yang S.-F., Yang J.-S., Lin C.-W. (2018). Sulforaphane suppresses oral cancer cell migration by regulating cathepsin S expression. Oncotarget.

[43] Hsin M.-C., Hsieh Y.-H., Wang P.-H., Ko J.-L., Hsin I.-L., Yang S.-F. (2017). Hispolon suppresses metastasis via autophagic degradation of cathepsin S in cervical cancer cells. Cell Death Dis..

[44] Syed D.N., Chamcheu J.-C., Khan M.I., Sechi M., Lall R.K., Adhami V.M., Mukhtar H. (2014). Fisetin inhibits human melanoma cell growth through direct binding to p70S6K and mTOR: findings from 3-D melanoma skin equivalents and computational modeling. Biochem. Pharmacol..

[45] Khan M.I., Adhami V.M., Lall R.K., Sechi M., Joshi D.C., Haidar O.M., Syed D.N., Siddiqui I.A., Chiu S.-Y., Mukhtar H. (2014). YB-1 expression promotes epithelial-to-mesenchymal transition in prostate cancer that is inhibited by a small molecule fisetin. Oncotarget.

[46] Li J., Gong X., Jiang R., Lin D., Zhou T., Zhang A., Li H., Zhang X., Wan J., Kuang G. (2018). Fisetin Inhibited Growth and Metastasis of Triple-Negative Breast Cancer by Reversing Epithelial-to-Mesenchymal Transition via PTEN/Akt/GSK3beta Signal Pathway. Front. Pharmacol..

